# Trans-oral anterior fundoplication: 5-year follow-up of pilot study

**DOI:** 10.1007/s00464-015-4142-9

**Published:** 2015-03-18

**Authors:** Aviel Roy-Shapira, Amol Bapaye, Suhas Date, Rajendra Pujari, Shivangi Dorwat

**Affiliations:** 1 Department of Surgery A, Soroka University Medical Center and the Faculty of the Health Sciences, Ben Gurion University, Beer Sheva, Israel; 2 Department of Digestive Diseases and Endoscopy, Deenanath Mangeshkar Hospital and Research Center, Pune, India

**Keywords:** GORD/GERD (Gastro-oesophageal reflux disease), Clinical papers/trials/research, G-I < endoscopy, GI < surgery

## Abstract

**Background:**

This is a report of an IRB-approved pilot study of 13 patients who received a trans-oral anterior partial fundoplication for the treatment of GERD using an ultrasound-guided, flexible surgical stapler. All patients had a history of PPI use, objective evidence of GERD, and no significant comorbidity. Under general anesthesia, a flexible stapler was passed trans-orally into the stomach and placed two or three quintuplets of titanium staples approximately 3 cm above the gastroesophageal junction. The stapler contains an ultrasonic range finder, video camera, and illuminator.

**Methods:**

Primary follow-up at 6 weeks included pH metrics, GERD-HRQL scores, and PPI use. The protocol allowed annual telephone interviews for the following 5 years to collect GERD-HRQL scores, PPI use, satisfaction with the procedure, and willingness to have the procedure again.

**Results:**

At 6 weeks, mean total acid exposure was significantly reduced, and 12/13 patients reduced GERD-HRQL scores by ≥50 %. Twelve of 13 patients had stopped daily GERD medications, and nine of 13 had stopped all GERD medications. Each year, 11 of the 13 patients could be reached with all 13 patients having at least 4-year follow-up. Throughout the follow-up period, GERD-HRQL scores were normal (<10) in all but one patient. All patients would agree to do the procedure again. The median satisfaction score is 8 (range 6–10) on a scale of 1–10. None reported dysphagia. At 1 year, 54 % of respondents (6/11) had eliminated PPI use, with another 27 % (3/11) taking a reduced dose. Combining respondents at 4 and 5 years to account for all patients, 54 % (7/13) had eliminated and another 23 % (3/13) reduced PPI use ≥50 %.

**Conclusion:**

At 5 years, the procedure remained effective as demonstrated by the improved quality of life and changes in PPI use. The results remained stable after the second year.

In this paper, we provide long-term follow-up data on a group of 13 subjects who participated in a pilot study of a new device (MUSE**™**, Medigus Ltd, Omer, Israel), which enables a single operator to trans-orally perform anterior fundoplication (Dor–Thal [1]) for the treatment of moderate-to-severe gastroesophageal reflux disease (GERD).

There is level 1a evidence that anterior fundoplication is as effective as a 360° fundoplication (Nissen) for symptom control in moderate-to-severe GERD [2]. Originally, both operations were done via a thoracotomy or a laparotomy, but today, both are generally performed laparoscopically with similar results [3, 4]. The new device was developed in order to make further progress in reducing the invasiveness of the surgical treatment of GERD, by providing a totally trans-oral standard anti-reflux operation.

The new method uses standard 4.8 mm “B” titanium surgical staples. In the process of the development, an ex vivo study demonstrated that a stapled anterior fundoplication had an anti-reflux effect similar to that of a 360 fundoplication, independent of supporting structures [5]. In addition, the safety and histologic tissue fusion of the stapled fundoplication was tested in survival studies on the swine model [6].

The results of the 6-week follow-up of this pilot study were used to support the initiation of a larger, multicenter pivotal study, the six-month results of which were recently published [7]. This report is the first publication of long-term outcome data of the trans-oral device.

## Patients and methods

Between March and October 2007, 15 men and women with moderate-to-severe GERD were enrolled in the study. All signed a detailed informed consent form (ICF). The form was written in English, translated to the local language (Marathi), and then translated back to English by an independent translator for verification. The ICF allowed the investigators or their designees to interview the patient annually for 5 years following the procedure. The study protocol was approved by the hospital’s institutional review board (IRB).

The inclusion and exclusion criteria are listed in Table 1. All patients were on high-dose PPI treatment, defined as twice the standard daily dosing, with incomplete clinical response, as measured by a well-validated questionnaire (Velanovich GERD health-related quality of life—GERD-HRQL) [8]. All had objective measures of GERD as indicated by 24-h acid exposure tests and symptom correlation or esophagitis. All had a dysfunctional lower esophageal high-pressure zone (LEHPZ) as determined by manometry, and none had gastric outlet obstruction.

**Table 1:** Inclusion and exclusion criteria

Inclusion criteria	Exclusion criteria
Age ≥ 18 years	BMI > 35
Objective evidence of GERD	Significant comorbidity
pH < 4 more than 7 % of time	Hiatal hernia >3 cm
pH < 4 between 4 and 7 % of time with >50 % correlation between symptoms and acid reflux events	Barrett’s esophagus Esophageal stricture Grade IV esophagitis
Esophagitis	
Ability to read and sign ICF	

Patients with significant comorbidity were excluded. Additional exclusion criteria were grade IV esophagitis, esophageal stricture, BMI >35, and hiatal hernia >3 cm.

**Fig. 1:**
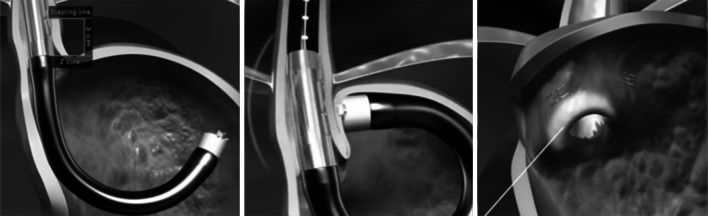
Trans-oral stapling procedure: Stapler positioned under direct visualization (*left*), Stapling placed ~3 cm cephalad to GEJ (*middle*); each stapling delivers a five titanium staples (*right*). Schematics courtesy of Medigus, Ltd

The procedure was conducted under general anesthesia in an endoscopy suite. Standard gastroscopy was performed to verify that no exclusion criteria have developed between screening and the procedure. An overtube was slid over the gastroscope and placed into the mid-esophagus. The stapling device was then inserted through the overtube into the stomach under direct visualization with an integrated video camera. The device was articulated to visualize and select the target stapling location. As the distal tip of the device touches the fundus, direct visualization is replaced by ultrasound to determine the distance between the distal tip and the staple cartridge, which is located in the shaft of the device. When an appropriate gap is achieved, five 4.8-mm titanium surgical staples (identical to the standard B-shaped staples used for gastroesophageal anastomoses) are fired simultaneously. The fundus was stapled at two or more locations about 3 cm cephalad to the gastroesophageal junction (GEJ), covering approximately 180 degrees of the esophagus (see Fig. 1).

Six weeks after the procedure, the patients were re-examined and underwent repeat endoscopy, ambulatory acid exposure test, and GERD-HRQL, and were questioned regarding adverse events, medication use, and GERD symptoms.

Patients were subsequently interviewed annually by phone. The structured interview consisted of repeating the GERD-HRQL questionnaire, medication use, general well-being, and satisfaction with the procedure.

Thirteen of the 15 enrolled patients had the full procedure and are included in the efficacy results. The procedure was abandoned in two patients prior to the placement of staples. One patient was a 20-year-old woman with a BMI of 18. The combined thickness of the walls of the stomach and esophagus, as measured by ultrasound, was too thin for safe stapling with 4.8-mm staples. A second patient was cancelled prior to the placement of the overtube due to a complication during the preliminary gastroscopy.

**Fig. 2:**
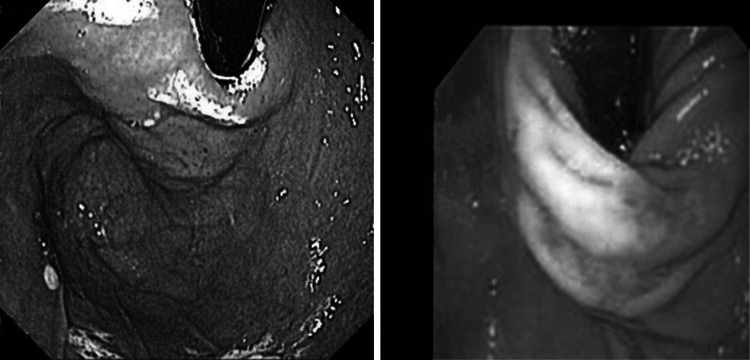
Retrograde view of trans-oral stapled anterior fundoplication (*left*) and laparoscopic fundoplication (*right*). Photograph on right courtesy of Prof. D. Watson, Adelaide, Australia

Demographic data for the treated patients are provided in Table 2. The fundus was stapled to the esophagus with two quintuplets in four patients and with three quintuplets in nine patients. On gastroscopic examination immediately after the procedure, the result was anatomically identical to a Dor–Thal anterior fundoplication (see Fig. 2).

**Table 2:** Patient demographic data

Number of patients enrolled	15
Patients receiving procedure	13
Age, years	
Median (range)	46 (23–64)
Gender	11 Male; 2 Female
Duration of GERD symptoms, years	
Median (range)	2.5 (0.5–20)
BMI (range)	18–31

There was one instance of benign pneumoperitoneum, which resolved spontaneously within 48 h. There were three additional adverse events in this series, all unrelated to the study device. One patient, a 65-year-old man had urinary retention and required catheterization. One patient was found to have a duodenal ulcer at 6 weeks. He was tested positive for H. Pylori. After treatment, H. Pylori was eradicated, and the ulcer healed. Another patient developed superficial thrombophlebitis at the site of IV line placement.

Baseline and 6-week efficacy data are provided in Table 3. The primary success criterion was at least 50 % improvement in off PPI GERD-HRQL scores. At 6 weeks, median (range) GERD-HRQL was reduced from 24 (10–38) at baseline to 5 (1–15). Twelve of 13 patients (92 %) had improved GERD-HRQL scores by more than 50 %. The remaining subject had a post-procedure score of 15 and a baseline score of 29 (48 % reduction).

**Table 3:** Baseline and 6-week results for GERD-HRQL and acid exposure

Result	Baseline	6 weeks
GERD-HRQL		
Mean ± SD	24.2 ± 6.9	6.5
Median	24	5
Range	10–38	1–15
Acid exposure (% time pH < 4)		
Mean ± SD	16.3 ± 13.6	8.5 ± 8.6
Median	11.0	5.0
Range	4.9–49.8	1.0–27.3

Mean total acid exposure was significantly reduced (*p* < 0.002, ranked sign test) from 16.3 to 8.6 (median 11.0 to 5.0). Seven of 13 patients (54 %) had normalized acid exposure as defined by percent of total time pH < 4 of 5 % or less.

Use of daily PPI was eliminated in 12/13 patients (92 %), with 10/13 (69 %) completely off of any PPIs. One patient was on a reduced daily dosage, one used PPIs on alternate days, and one claimed to use PPIs only occasionally. One patient reported taking an occasional H2 receptor blocker, but no PPI.

Annual follow-up data are provided in Table 4. Each year during the annual phone interviews, at least 11 patients could be located, but not always the same 11. However, all 13 patients had at least 4 years of follow-up.

**Table 4:** Annual follow-up

Follow-up	6 weeks	1 year	2 year	3 year	4 year	5 year	4 or 5 year
n	13/13	11/13	11/13	11/13	11/13	11/13	13/13
GERD-HRQL > 50 %	12	10	11	10	11	10	12
Daily PPI	1	2	3	3	3	3	3
>50 % reduction	2	3	3	3	3	3	3
No	10^a^	6	5	5	5	5	7
Dysphagia	0	0	0	0	0	0	0
Gas Bloat	0	0	0	0	0	0	0
Do again	All	All	All	All	All	All	All

^a^One patient reported occasional H2 receptor blocker use

All patients but one had at least 50 % reduction in their pre-procedure GERD-HRQL score on all follow-up interviews. The exception was the patient whose preoperative score was 29. The score was 14 at years 2 and 4 (>50 % reduction), and 15 at years 1, 3, and 5. This was the only patient who reported a non-normal (>10) score at any interview.

At 1 year, only two of 11 (18 %) patients used daily PPI, and 54 % of respondents (6/11) had eliminated PPI use, with another 27 % (3/11) taking a reduced dose. At 2 years post-procedure, three of 11 patients were using daily PPI (one at the baseline dose and two at half the baseline dose). One patient was using PPI only occasionally after heavy meals. From the second year onward, no changes in PPI usage were reported. Combining responses at 4 and 5 years to account for all patients, 64 % (7/13) of patients had eliminated and another 23 % (3/13) reduced PPI use by 50 % or more.

At every follow-up interview, all patients were satisfied with the operation, and all would have agreed to undergo it again.

Some of the patients reported gas bloat after heavy meals during the first month, but none reported such experiences later than 6 weeks.

## Discussion

Herein, we report the long-term results of the long-term follow-up of a less invasive method for creating anterior fundoplication.

Between the 6-week visit and the 2-year interview, there was a small increase in the number of patients taking PPI. However, the patients remained satisfied and free of symptoms, whereas before the procedure, all were symptomatic on their daily dosage. This observation is similar to that observed following Nissen fundoplication. In a frequently quoted study, Spechler et al. [9] found that in 10 years, >60 % of patients eventually return to PPI treatment; however, they remain asymptomatic and satisfied with the results of the operation.

A procedure failure occurred in a very thin woman. The combined thickness of the esophageal and the gastric walls were outside the operating range of the device. An ultrasound transducer at the distal tip of the device allows precise calculation of the tissue thickness, and the user cannot staple outside of a predetermined range (too thick or too thin). The range is based on the 4.8 mm staple dimensions. It is possible that using 3.8-mm staples (Autosuture™ “blue”) would have permitted safe stapling, but the device does not support their use. Although one subject with a BMI of 18 had a successful procedure, the instructions for use (IFU) of the marketed version of the device count a BMI of 20 or less as a relative contraindication.

Urinary retention after general anesthesia is not uncommon in 65-year-old men with enlarged prostate, and patients, just as was found in this case, usually respond to catheterization and medications [10].

The duodenal ulcer discovered at the 6-week gastroscopy was asymptomatic. The patient was H. Pylori positive and most likely developed the ulcer because the procedure effectively eliminated the symptoms of GERD. As long as the patient was on PPI drugs, he was protected from developing a peptic ulcer. At the 2-year follow-up, this patient went back to PPI for dyspepsia, not for reflux symptoms. This may be indicative of re-colonization with H. Pylori rather than failure of the procedure. While re-infection is rare in developed environments, in developing countries, it is quite common, particularly after the first year and is estimated at 12 % annually [11].

The new trans-oral device uses standard surgical staples instead of sutures for tissue approximation. Our findings are in agreement with the well-known observations that staples of this type are as effective and durable as sutures for approximating tissues in the GI tract [12].

The second major difference between the laparoscopic and the trans-oral approach presented here is that the latter was not intended to treat a sliding hiatal hernia, if present, and, for safety considerations, is limited to patients with HH ≤ 3 cm. Although over half the patients with GERD have a concomitant HH, a significant number, particularly among patients <50 years old, do not [13]. Moreover, the need to repair small HH’s is not clear. Richardson et al. [14] demonstrated that fundoplication, either partial or complete, was an independent complete reflux barrier, and failure of the crural repair 6 months after LF does not usually result in recurrent symptoms [15].

This pilot study was a precursor to a larger multicenter pivotal trial [7]. The pilot study used a non-commercial version of the MUSE system. The commercial version is similar, but has a streamlined user interface and a lighter construction.

In this series, as well as in the subsequent larger study, all the procedures were performed by or under supervision of experts in advanced endoscopy or in the use of the device. Whether non-experts can achieve similar results remains an open question.

## Conclusion

Although the number of pilot patients was small, the long-term pilot results point to a trend. The data suggest that after 2 years, the results of a stapled trans-oral anterior fundoplication with the endoscopic stapler (MUSE™, Medigus Ltd) remain stable and are similar to those reported for laparoscopic approach. Consequently, it appears to offer a safe and effective alternative to the more invasive laparoscopic route, but for the present, should be restricted to physicians with proven expertise in advanced endoscopic methods. The long-term follow-up of larger studies, such as the pivotal study that led to FDA clearance, is needed to confirm these results.
